# Serum Creatinine as an Independent Predictor of Moderate to Severe Fibrosis in Chinese American Non-obese Metabolic Dysfunction-Associated Steatotic Liver Disease

**DOI:** 10.7759/cureus.61116

**Published:** 2024-05-26

**Authors:** Michael Sun, Vincent J Yao, Aivi A Rahman, Kevin Liu, Saud Rehman, Amber Sun, Alan C Yao

**Affiliations:** 1 College of Agriculture and Life Sciences, Cornell University, Ithaca, USA; 2 College of Medicine, Sophie Davis Biomedical Education Program, City University of New York (CUNY) School of Medicine, New York, USA; 3 College of Medicine, Sophie Davis Biomedical Education Program, City University of New York (CUNY) School of Medicine,, New York, USA; 4 College of Arts and Sciences, New York University, New York, USA; 5 College of Liberal Arts and Sciences, Donald and Barbara Zucker School of Medicine at Hofstra/Northwell, Hempstead, USA; 6 Gastroenterology and Hepatology, Long Island Jewish Medical Center, Northwell Health, New York, USA

**Keywords:** metabolic dysfunction-associated steatotic liver disease (masld), fibrosis, risk factor, creatinine, asian

## Abstract

Background: Metabolic dysfunction-associated steatotic liver disease (MASLD) is closely linked to the obesity epidemic. However, non-obese MASLD (body mass index [BMI] < 25 kg/m^2^ for Asians) is not uncommon, especially among Asian American populations. Preliminary research has demonstrated sarcopenia, a muscle-wasting syndrome, to be a major risk factor for non-obese Chinese MASLD. This study examined serum creatinine (SCr), a sarcopenia biomarker, and other prominent MASLD biomarkers for their ability to predict moderate to severe fibrosis (≥7.5 kPa or ≥F2 fibrosis) in the Chinese American MASLD population.

Methods: A total of 296 Chinese American MASLD patients were categorized by BMI and fibrosis severity. As per World Health Organization guidelines for Asians, we identified obese MASLD (BMI ≥ 25 kg/m^2^) in 191 subjects (64.5%) and non-obese MASLD (BMI < 25 kg/m^2^) in 105 subjects (35.5%). Multivariate logistic regressions were performed to ascertain which biomarkers served as independent predictors of ≥F2 fibrosis. Wilcoxon signed-rank tests were conducted to compare MASLD cohorts (stratified by gender) and the healthy adult population on SCr distribution.

Results: The obese MASLD cohorts had higher rates of ≥F2 fibrosis and type 2 diabetes mellitus compared to their older, non-obese counterparts. For obese MASLD patients, higher age (P < 0.05), increased BMI (P < 0.01), increased AST (P < 0.05), and decreased platelets (P < 0.05) independently predicted ≥F2 fibrosis. For non-obese MASLD patients, lowered SCr (P < 0.05) levels served as the main predictor of ≥F2 fibrosis. Female MASLD patients had markedly lower SCr distributions (P < 0.001) compared to the healthy female population, with 26.8% having SCr levels below the normal range.

Conclusions: In summary, SCr was the predominant predictor of moderate to severe fibrosis in non-obese Chinese American MASLD patients. The high rate of decreased SCr levels in Chinese American MASLD women suggests that this population may be at higher risk for muscle mass loss, which can lead to liver fat accumulation.

## Introduction

Metabolic dysfunction-associated steatotic liver disease (MASLD) is currently the most widespread liver disease, with a global prevalence of 25% [1]. If MASLD is not monitored properly, it can progress to metabolic dysfunction-associated steatohepatitis (MASH), which is associated with a higher risk of hepatocellular carcinoma and cirrhosis [2]. In recent years, the increased prevalence of obesity and MASH-induced cirrhosis has propelled MASLD to become one of the most common indications for liver transplantation [3].

While the majority of MASLD cases have been linked to obesity, the rate of non-obese MASLD (defined by a body mass index [BMI] ≤ 30 kg/m^2^ in Caucasians and BMI ≤ 25 kg/m^2^ in Asians) is evidently significant [4]. A 2020 retrospective cohort study investigating the United States (US) MASLD population found 70.3% to be obese and 29.7% to be non-obese, with most non-obese patients being of Asian descent [5]. A 2019 meta-analysis reported that within the Asian MASLD population, the rate of non-obese MASLD was up to 40% [6].

The Asia-Pacific guidelines highlight Asians as more susceptible to non-obese MASLD than Caucasians and other races. Different body composition in muscle and fat tissue, predisposition to type 2 diabetes mellitus (T2DM), and high prevalence of the variant allele rs738409 of PNPLA3 (a MASLD risk factor present in up to 19% of the Chinese population) support the notion that Asian MASLD, particularly the non-obese form, may have a distinct pathophysiology [7,8].

Interestingly, sarcopenia, a muscle atrophy condition with higher prevalence in the older Chinese population, was recently shown to be a strong risk factor (RF) for non-obese MASLD [9,10]. Sarcopenic Chinese patients were observed to have a higher risk of non-obese MASLD compared to non-sarcopenic patients, regardless of visceral adiposity or other metabolic RFs. Furthermore, the adverse effect of sarcopenia on non-obese Chinese MASLD was not influenced by alternative metabolic RFs [11].

Serum creatinine (SCr) was observed to be a reasonable biomarker for sarcopenia in individuals with normal renal function. For this group, sarcopenia was often the cause of below-normal SCr levels [12]. The direct correlation between SCr and skeletal muscle mass has allowed the former to be used as an indicator of muscle mass [13]. In addition, a 2015 analysis of the Korea National Health and Nutrition Examination Survey demonstrated low SCr to be independently associated with both appendicular muscle mass loss and bone mineral density loss in older adults [14].

Despite Asian Americans accounting for nearly half the non-obese MASLD cases in the US, MASLD research about this subgroup remains scarce [15]. While Asian Americans have often been lumped with other Asians in analyses of MASLD cohorts, it is important to assess them separately as Asian Americans may possess distinct lifestyle factors and genetic signatures. This study investigated SCr and other relevant biomarkers for their potential to predict moderate to severe fibrosis in the Chinese American MASLD population. Moreover, we sought to compare the SCr distributions of our Chinese American MASLD cohorts with that of the healthy adult population [16]. We hypothesized that lower SCr, reflecting low skeletal muscle mass, maybe a potential point of interest with regard to Chinese American MASLD.

## Materials and methods

Inclusion criteria

Chinese American patients above the age of 18 with a MASLD diagnosis were included in the study. Per the World Health Organization guidelines for Asians, patients with BMI ≥ 25 kg/m^2^ were classified as obese, while those with BMI < 25 kg/m^2^ were classified as non-obese [4]. MASLD diagnoses were based on a combination of elevated alanine aminotransferase (ALT) levels (≥40 IU/mL for men and ≥31 IU/mL for women), apparent steatosis detected via hepatic ultrasounds, abnormal liver stiffness measurement (LSM) results obtained by FibroScan® (Echosens, Westborough, MA), and exclusion of other causes of liver disease and steatosis in the absence of significant alcohol consumption (daily alcohol intake of >20g) [17]. FibroScan®, a form of transient elastography, is a reliable, non-invasive alternative to liver biopsy [18].

Exclusion criteria

Chinese American patients under the age of 18 were excluded from the study. Patients with a history of bariatric surgery, significant alcohol consumption (daily alcohol intake of >20g), polycystic ovarian syndrome, and type 1 diabetes mellitus (T1D), which was diagnosed based on elevated hemoglobin A1c and positive autoantibody testing to T1D, were excluded. Other underlying liver diseases with the potential to affect liver fibrosis were also excluded, including drug-induced liver disease, alcoholic liver disease, autoimmune hepatitis, primary biliary cirrhosis, α1-antitrypsin deficiency, hemochromatosis, Wilson’s disease, biliary obstruction, and chronic hepatitis B and C. Furthermore, diseases and conditions that could impact SCr (not including MASLD) were excluded, including syndrome of inappropriate antidiuretic hormone secretion, nephrotic syndrome, augmented renal clearance, chronic kidney disease (estimated glomerular filtration rate of >60 mL/min/1.73 m^2 ^for over three months), diuretic use, diarrhea, vegetarian lifestyle, pregnancy, hemiplegia, paraplegia, and other neuromuscular disorders [12]. Patients who lacked a complete biomarker profile within one year of recorded FibroScan® readings were excluded. FibroScan results with interquartile range (IQR) ≥ 0.3 kPa were also excluded.

Study procedure

Chinese American MASLD patients from two gastrointestinal clinics were categorized into non-obese and obese groups to account for the impact of BMI on MASLD pathophysiology. Collected biomarkers were then assessed for their ability to predict moderate to severe fibrosis (≥7.5 kPa). The non-obese and obese groups were then further categorized into the non-obese male (NM), obese male (OM), non-obese female (NF), and obese female (OF) groups to account for the impact of gender on SCr. The SCr distributions of these four cohorts were then compared to those of the healthy adult population [16]. The biomarker profiles of these four MASLD groups were also recorded.

Biomarker and LSM values were collected from laboratory reports and FibroScan® readings, respectively, from 2016 to 2021. The data values of each patient were obtained within a one-year time frame. For the FibroScan® procedure, an M/XL probe was positioned perpendicular to the coated skin after the intercostal space was identified. Ten valid scans were then taken with an interquartile range of <0.3 kPa. Following standard clinical protocol, LSM values were recorded in patients who met a fasting condition at least four hours before the examination. Patient age, gender, race, and BMI were recorded during annual physical examinations. MASLD patients were classified based on fibrosis severity into either the F0-F1 or ≥F2 groups, with 7.5 kPa as the cutoff for F2. F0-F1 specified mild to minimal fibrosis, F2 specified moderate fibrosis, F3 specified advanced fibrosis, and F4 specified cirrhosis [19].

An affiliated institutional review board (IRB) was unavailable as this retrospective cohort study was conducted at a private practice gastroenterology clinic in New York. This study was administered without any patient interaction or intervention and posed minimal risk to participants. Patient names were converted into anonymous codes for confidentiality, and the data consisted solely of existing records.

Statistical analyses

A Kolmogorov-Smirnov test was performed to ascertain the normality of each variable. Normally distributed variables were expressed as mean ± standard deviation, while non-normal variables were expressed as medians with interquartile ranges. Two multivariate logistic regression models were constructed to determine which variables were independent predictors of moderate to severe fibrosis (≥7.5 kPa) in the non-obese and obese groups. All baseline characteristics, excluding aspartate aminotransferase (AST)/ALT, total cholesterol (TC)/HDL cholesterol, and triglycerides (TG)/HDL cholesterol, were categorized as covariates for these multivariate logistic regressions. The findings were presented as odds ratios with 95% confidence intervals.

As the SCr values of our Chinese American MASLD sample were not normally distributed, we conducted a non-parametric statistical test. Four separate Wilcoxon signed-rank tests were performed to compare the SCr distributions of these four cohorts (NM, OM, NF, and OF) with the SCr distribution of the healthy adult population [16]. All statistical calculations were performed using Python 3.11.9 (Python Software Foundation, Wilmington, DE) with the SciPy 1.11.2 (SciPy Community), scikit-learn 1.4.0 (Scikit-learn Community), and statsmodel 0.14.1 (Statsmodel Community) packages. P-values ≤ 0.05 (two-tailed) were noted to be statistically significant.

## Results

Baseline characteristics

We reviewed the records of 4,609 MASLD patients. Based on our inclusion and exclusion criteria, 296 patients were incorporated into the analysis. Patients were stratified based on BMI and gender into four groups: 44 NM, 114 OM, 61 NF, and 77 OF. Groups with the same BMI classification (NM/NF and OM/OF) were similar in both age and BMI. For NM, four were identified with T2DM (9.1%) and one with ≥7.5 kPa (2.3%). For OM, 33 were identified with T2DM (28.9%) and 25 with ≥7.5 kPa (21.9%). For NF, nine were identified with T2DM (14.8%) and eight with ≥7.5 kPa (13.1%). For OF, 17 were identified with T2DM (22.1%) and 11 with ≥7.5 kPa (14.3%). The median SCr of the NM (79.1μmol/L) and OM (80μmol/L) cohorts were noted to be lower than the NF (60.1μmol/L) and OF (61.9μmol/L) cohorts (Table 1).

**Table 1:** Baseline characteristics of obese and non-obese Chinese American MASLD patients Table data is presented as mean ± SD, counts, or medians and interquartile ranges. *Percent of patients with moderate to severe fibrosis. SBP: Systolic blood pressure; DBP: Diastolic blood pressure; BUN: Blood urea nitrogen; e-GFR: Estimated glomerular filtration rate; AST: Aspartate aminotransferase; ALT: Alanine aminotransferase; TC: Total cholesterol; TG: Triglycerides; HDL: High-density lipoproteins; LDL: Low-density lipoproteins; WBC: White blood cell count; T2DM: Type II diabetes mellitus; LSM: Liver stiffness measurement; MASLD: Metabolic dysfunction-associated steatotic liver disease.

Characteristics	Non-obese male (n = 44)	Obese male (n = 114)	Non-obese female (n = 61)	Obese female (n = 77)
Age	58.7 ± 14.5	53.8 ± 12.9	60.4 ± 9.2	56.1 ± 13.6
SBP (mmHg)	120 (110–130)	121 (110–131)	110 (105–128)	122 (118–130)
DBP (mmHg)	72 (70–80)	80 (70–84)	70 (70–80)	75 (70–80)
Body mass index (kg/m^2^)	23.9 (22.1–24.7)	28.2 (26.9–31.0)	23.4 (22.1–24.4)	28.9 (26.8–31.4)
Albumin (g/dL)	4.6 (4.4–4.7)	4.6 (4.4–4.7)	4.6 (4.4–4.7)	4.5 (4.3–4.6)
Glucose (mg/dL)	98 (93–105)	101 (91–118)	98 (91–109)	102 (94–114)
BUN (mg/dL)	15.5 ± 3.5	15.9 ± 3.6	14.5 ± 3.1	14.9 ± 4.6
Serum creatinine (µmol/L)	79.1 (71.2-88.4)	80 (73.4-91.3)	60.1 (54.8-66.3)	61.9 (53.1-68.1)
e-GFR (mL/min/1.73 m^2^)	90.6 ± 16.4	90.1 ± 17.3	90.8 ± 22.9	94.0 ± 16.5
Bilirubin (mg/dL)	0.7 (0.5–0.9)	0.6 (0.5–0.9)	0.5 (0.4–0.6)	0.5 (0.3–0.6)
AST (U/L)	24 (17–28)	24 (19–33)	24 (20–30)	26 (19–33)
ALT (U/L)	27 (17–35)	36 (21–53)	24 (19–35)	33 (20–49)
TC (mg/dL)	181 ± 38	183 ± 35	198 ± 33	195 ± 36
TG (mg/dL)	150.0 ± 85.9	162.1 ± 77.5	151.4 ± 72.3	152.6 ± 53.6
HDL cholesterol (mg/dL)	48 (40–58)	45 (38–49)	54 (48–65)	50 (44–58)
LDL cholesterol (mg/dL)	100.3 ± 37.0	107.3 ± 32.6	109.9 ± 29.1	114.8 ± 33.1
WBC (/µl)	5.5 ± 1.5	6.6 ± 1.6	5.8 ± 1.6	6.3 ± 1.7
Platelets (/µl)	204.8 ± 61.8	216.7 ± 57.8	242.4 ± 52.2	248.9 ± 64.9
T2DM (Yes/No)	4/40	33/81	9/52	17/60
LSM (kPa)	4.8 (4.0–5.4) (2.3%)*	5.4 (4.4–7.3) (21.9%)*	4.4 (3.8–5.6) (13.1%)*	5.4 (4.2–6.6) (14.3%)*
AST/ALT	0.9 ± 0.3	0.8 ± 0.3	1.0 ± 0.3	0.9 ± 0.3
TC/HDL cholesterol	3.7 (3.0–4.5)	4.1 (3.5–4.9)	3.6 (3.0–4.1)	3.8 (3.4–4.4)
TG/HDL cholesterol	2.3 (1.5–4.6)	3.4 (2.5–4.4)	2.7 (1.6–3.6)	3.0 (2.2–3.9)

Multivariate analysis

We performed two separate multivariate logistic regression analyses to determine the independent predictors of ≥F2 fibrosis in the obese and non-obese groups. All variables in Table 1 (excluding AST/ALT, TC/HDL cholesterol, and TG/HDL cholesterol) were accounted for in the analysis. In obese MASLD patients, higher age (P < 0.05; OR, 1.094; β, 0.090; 95% CI, 0.008-0.173), increased BMI (P < 0.01; OR, 1.286; β, 0.252; 95% CI, 0.068-0.435), increased AST (P < 0.05; OR, 1.057; β, 0.055; 95% CI, 0.001-0.110), and decreased platelets (P < 0.05; OR, 0.990; β, -0.010; 95% CI, -0.020-0.000) were independent predictors of ≥F2 fibrosis (Table 2). In non-obese MASLD patients, decreased SCr (P < 0.05; OR, 0.883; β, -0.125; 95% CI, -0.238-0.011) was the only independent predictor of ≥F2 fibrosis (Table 3).

**Table 2:** Risk factors for moderate to severe fibrosis in obese Chinese American MASLD patients β: Regression coefficient; SE: Standard error of regression coefficient; SBP: Systolic blood pressure; DBP: Diastolic blood pressure; BUN: Blood urea nitrogen; e-GFR: Estimated glomerular filtration rate; AST: Aspartate aminotransferase; ALT: Alanine aminotransferase; TG: Triglycerides; TC: Total cholesterol; HDL: High-density lipoprotein; LDL: Low-density lipoprotein; WBC: White blood cell count; T2DM: Type II diabetes mellitus; MASLD: Metabolic dysfunction-associated steatotic liver disease.

Characteristics	β	SE	Z	P	OR	[0.025]	[0.975]
Age	0.090	0.042	2.143	0.032	1.094	0.008	0.173
SBP	-0.048	0.027	-1.756	0.079	0.953	-0.102	0.006
DBP	-0.014	0.040	-0.335	0.738	0.987	-0.093	0.066
Body mass index	0.252	0.094	2.686	0.007	1.286	0.068	0.435
Albumin	1.875	1.129	1.661	0.097	6.522	-0.338	4.088
Glucose	0.002	0.009	0.257	0.797	1.002	-0.015	0.019
BUN	0.031	0.076	0.406	0.685	1.032	-0.119	0.181
Serum creatinine	-0.010	0.032	-0.301	0.763	0.990	-0.072	0.053
e-GFR	0.030	0.043	0.701	0.483	1.031	-0.055	0.115
Bilirubin	0.780	0.757	1.031	0.303	2.182	-0.703	2.263
AST	0.055	0.028	2.005	0.045	1.057	0.001	0.110
ALT	0.008	0.015	0.565	0.572	1.008	-0.021	0.038
TG	0.036	0.089	0.400	0.689	1.036	-0.139	0.210
TC	-0.006	0.018	-0.321	0.748	0.994	-0.041	0.029
HDL cholesterol	-0.098	0.096	-1.021	0.307	0.907	-0.286	0.090
LDL cholesterol	-0.061	0.090	-0.683	0.495	0.940	-0.237	0.115
WBC	0.273	0.191	1.435	0.151	1.314	-0.100	0.647
Platelets	-0.010	0.005	-2.059	0.040	0.990	-0.020	0.000
T2DM	0.151	0.754	0.201	0.841	1.163	-1.327	1.629

**Table 3:** Risk factors for moderate to severe fibrosis in non-obese Chinese American MASLD patients β: Regression coefficient; SE: Standard error of regression coefficient; SBP: Systolic blood pressure; DBP: Diastolic blood pressure; BUN: Blood urea nitrogen; e-GFR: Estimated glomerular filtration rate; AST: Aspartate aminotransferase; ALT: Alanine aminotransferase; TG: Triglycerides; TC: Total cholesterol; HDL: High-density lipoprotein; LDL: Low-density lipoprotein; WBC: White blood cell count; T2DM: Type II diabetes mellitus; MASLD: Metabolic dysfunction-associated steatotic liver disease.

Characteristics	β	SE	Z	P	OR	[0.025]	[0.975]
Age	0.051	0.072	0.701	0.483	1.052	-0.091	0.193
SBP	-0.053	0.050	-1.060	0.289	0.948	-0.151	0.045
DBP	-0.007	0.093	-0.070	0.944	0.993	-0.189	0.176
Body mass index	0.796	0.488	1.632	0.103	2.216	-0.160	1.752
Albumin	-0.144	2.357	-0.061	0.951	0.866	-4.762	4.475
Glucose	0.036	0.029	1.221	0.222	1.036	-0.022	0.093
BUN	0.041	0.152	0.268	0.789	1.041	-0.257	0.338
Serum creatinine	-0.125	0.058	-2.157	0.031	0.883	-0.238	-0.011
e-GFR	-0.079	0.042	-1.888	0.059	0.924	-0.161	0.003
Bilirubin	0.742	1.601	0.465	0.642	2.105	-2.394	3.882
AST	0.059	0.102	0.574	0.566	1.060	-0.142	0.259
ALT	0.003	0.045	0.059	0.953	1.003	-0.086	0.092
TG	-0.967	0.701	-1.379	0.168	0.380	-2.341	0.407
TC	0.185	0.139	1.328	0.184	1.203	-0.088	0.458
HDL cholesterol	0.976	0.708	1.378	0.168	2.654	-0.412	2.364
LDL cholesterol	0.973	0.700	1.390	0.164	2.646	-0.398	2.344
WBC	0.435	0.386	1.127	0.260	1.546	-0.322	1.193
Platelets	-0.019	0.014	-1.334	0.182	0.981	-0.046	0.009
T2DM	-2.502	3.152	-0.794	0.427	0.082	-8.681	3.676

Serum creatinine distribution

Wilcoxon signed-rank tests were used to compare the four MASLD cohorts (NM, OM, NF, and OF) with the healthy adult population on SCr distribution [16]. Both female cohorts, NF (t = 368.0, P < 0.001) and OF (t = 576.5, P < 0.001), had significantly different SCr distributions compared to the healthy female population (Table 4). The NF and OF groups also had lower SCr medians and interquartile ranges (IQR) compared to the healthy female population (66.0 µmol/L [59.1-73.8 µmol/L]). No relevant differences in SCr distribution were observed between the healthy male population and the male cohorts, NM (t = 329.0, P = 0.082) and OM (t = 2832, P = 0.511) (Table 4). The NM and OM groups had similar SCr medians and IQRs compared to the healthy male population (81.6 µmol/L [74.7-88.6 µmol/L]) [16].

**Table 4:** Wilcoxon signed-rank test results comparing MASLD cohorts with the healthy adult population on serum creatinine distribution *Statistically significant results (P < 0.05). IQR: Interquartile range; MASLD: Metabolic dysfunction-associated steatotic liver disease.

MASLD cohort	P	t	Median (µmol/L)	IQR (µmol/L)
Non-obese male (n = 44)	0.082	329.0	79.1	(71.2-88.4)
Obese male (n = 114)	0.511	2832	80.0	(73.4-91.3)
Non-obese female (n = 61)	<0.001*	368.0	60.1	(54.8-66.3)
Obese female (n = 77)	<0.001*	576.5	61.9	(53.1-69.1)

**Figure 1:**
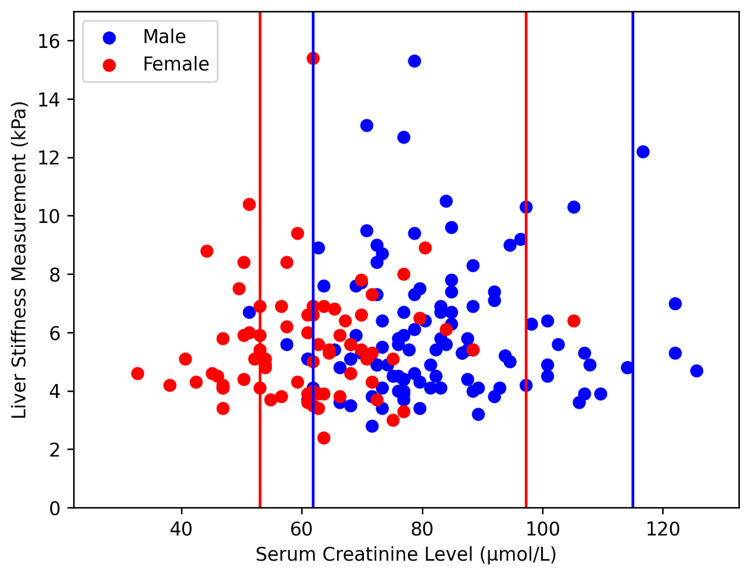
Serum creatinine values of obese Chinese American MASLD patients The blue and red vertical lines denote the normal serum creatinine ranges for men and women, respectively. MASLD: Metabolic dysfunction-associated steatotic liver disease.

**Figure 2:**
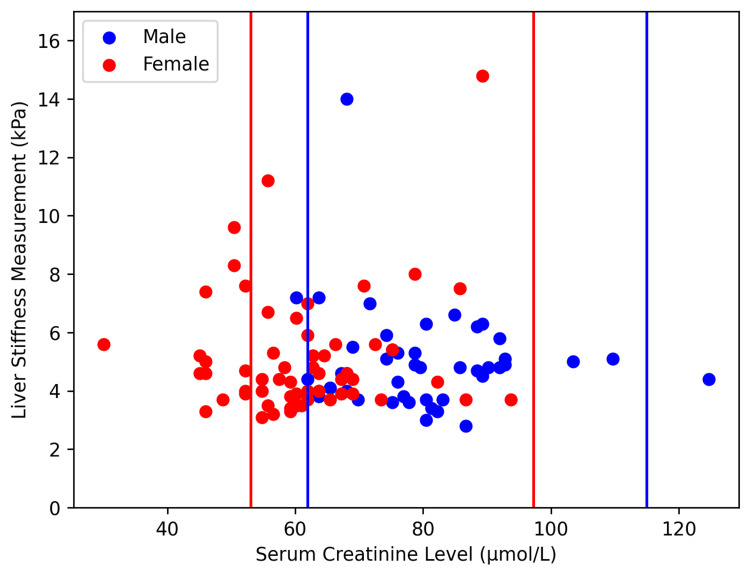
Serum creatinine values of non-obese Chinese American MASLD patients The blue and red vertical lines denote the normal serum creatinine ranges for men and women, respectively. MASLD: Metabolic dysfunction-associated steatotic liver disease.

To visualize the SCr distributions of the four MASLD cohorts, two scatterplots stratified by BMI were constructed (Figures 1, 2). The normal ranges of SCr, 61.9-114.9 µmol/L for men and 53-97.2 µmol/L for women, are denoted by blue and red vertical lines, respectively [20]. More than 20% of women in both the NF (14/61 or 23.9%) and OF (23/77 or 29.9%) groups had SCr values below the normal range. Only one female (1/77 or 1.3%), part of the OF cohort, had an SCr value above the normal range. For males, the NM cohort had two (4.5%) individuals under the SCr normal range and one (2.3%) individual over the SCr normal range. The OM group had seven (6.1%) under the SCr normal range and five (4.4%) over the SCr normal range (Figures 1, 2).

## Discussion

Despite the growing interest in non-obese MASLD, Asian American MASLD (comprising 48.7% of US non-obese MASLD cases) remains poorly understood due to the frequent pooling of Asian populations in MASLD analyses [15]. We therefore conducted the current study, with special attention paid to SCr (a biomarker of interest), to better characterize Chinese American MASLD. Our study found the obese Chinese American MASLD cohorts (OM and OF) to have a higher prevalence of moderate to severe fibrosis and T2DM compared to their older non-obese counterparts (NM and NF) (Table 1). All four MASLD cohorts lacked markedly elevated ALT levels; however, this trend was observed in a 2022 meta-analysis on Asian MASLD, which noted 85.7% of males and 60.7% of females to have normal ALT [21]. The NF and OF cohorts had noticeably lower SCr parameters compared to the NM and OM groups, which aligns with previous research reporting males to have higher basal levels of creatinine compared to females (Table 1) [22].

Interestingly, our multivariate analysis demonstrated decreased SCr to be the main predictor of ≥F2 fibrosis in the non-obese MASLD cohort (Table 2). Further non-parametric testing revealed significant differences in SCr distribution between our female MASLD cohorts and the healthy female population (Table 4). More specifically, the NF and OF cohorts had relatively high rates of below-normal SCr levels (Figures 1, 2). SCr is a known biomarker for muscle mass as 90% of its precursor, creatine phosphate, rests in muscle tissue. Moreover, low SCr levels have been an acceptable indicator for sarcopenia in individuals with normal kidney function [12]. Although several other conditions may lower SCr levels, they were disqualified from the study via the exclusion criteria. Our findings are consistent with previous data demonstrating that Chinese MASLD women have considerably lower muscle mass than the normal range [23].

A growing body of evidence suggests that the presence of sarcopenia significantly increases fibrosis risk in MASLD patients [24,25]. A 2017 prospective cohort study reported sarcopenia to increase fibrosis development with a relative risk of 2.05 [24]. Another 2021 Korean population-based study reported more significant fibrosis in sarcopenic MASLD patients than in their non-sarcopenic counterparts [25]. The link between MASLD severity and sarcopenia may be due to the role of skeletal muscle in insulin-mediated glucose absorption. Muscular atrophy reduces the cellular targets for insulin activity, inducing glucose intolerance and stimulating gluconeogenesis, which then accelerates muscle wasting and protein catabolism, decreasing SCr levels in the process [26]. Furthermore, as insulin resistance progresses, lipolysis rates increase, prompting the generation of free fatty acids (FFA), which are then stored by both muscle and liver tissue [27]. The insulin-resistance-mediated muscular atrophy then further amplifies FFA exposure and uptake by the liver as less muscle is available to absorb FFAs [28]. However, the overlapping pathophysiology of MASLD and sarcopenia makes it difficult to determine whether sarcopenia is an RF for MASH or a complication of it.

While a sarcopenia diagnosis should be made with measurements of muscle mass and function, SCr remains a key biomarker in the screening for sarcopenia [12]. Thus, physicians may find it beneficial to screen for sarcopenia when treating Chinese American MASLD patients.

Limitations

While we covered prominent MASLD RFs such as T2DM, dyslipidemia, and obesity in our analysis, the lack of data regarding other RFs, such as insulin resistance and visceral adiposity, is a major limitation [29]. We also acknowledge that the lack of a liver biopsy is a core limitation. Liver biopsy is an expensive and invasive procedure that cannot be reliably obtained for every patient [17]. Thus, the use of hepatic ultrasonography and transient elastography to diagnose and monitor MASLD reflects current clinical practice. Furthermore, the number of male subjects with non-obese MASLD was low; so, enlargement of the sample size is required for a multivariate analysis stratified by gender. As our cohort consisted only of Chinese American subjects, our results may not be applicable to other populations.

## Conclusions

This study found lowered SCr levels to be the primary predictor of moderate to severe fibrosis in non-obese Chinese American MASLD patients. SCr levels were also significantly lower in the NF and OF cohorts compared to the healthy female population, which suggests that Chinese American MASLD women may be at higher risk for lower muscle mass. Future research should investigate whether SCr levels can serve as a simple laboratory index to ascertain fibrosis severity in the Asian MASLD population. To better understand the underlying mechanisms behind SCr’s ability to predict advanced fibrosis, further studies are needed to discern the associations between SCr, sarcopenia, and Chinese American MASLD.

## References

[1] Younossi ZM, Koenig AB, Abdelatif D, Fazel Y, Henry L, Wymer M (2016). Global epidemiology of nonalcoholic fatty liver disease-meta-analytic assessment of prevalence, incidence, and outcomes. Hepatology.

[2] Chandrakumaran A, Siddiqui MS (2020). Implications of nonalcoholic steatohepatitis as the cause of end-stage liver disease before and after liver transplant. Gastroenterol Clin North Am.

[3] Battistella S, D'Arcangelo F, Grasso M (2023). Liver transplantation for non-alcoholic fatty liver disease: indications and post-transplant management. Clin Mol Hepatol.

[4] Weir CB, Jan A (2024). BMI Classification Percentile and Cut Off Points. BMI Classification Percentile And Cut Off Points.

[5] Zou B, Yeo YH, Nguyen VH, Cheung R, Ingelsson E, Nguyen MH (2020). Prevalence, characteristics and mortality outcomes of obese, nonobese and lean NAFLD in the United States, 1999-2016. J Intern Med.

[6] Li J, Zou B, Yeo YH (2019). Prevalence, incidence, and outcome of non-alcoholic fatty liver disease in Asia, 1999-2019: a systematic review and meta-analysis. Lancet Gastroenterol Hepatol.

[7] Wong VW, Chan WK, Chitturi S (2018). Asia-pacific working party on non-alcoholic fatty liver disease guidelines 2017-part 1: definition, risk factors and assessment. J Gastroenterol Hepatol.

[8] Lee GH, Phyo WW, Loo WM (2020). Validation of genetic variants associated with metabolic dysfunction-associated fatty liver disease in an ethnic Chinese population. World J Hepatol.

[9] Wu X, Li X, Xu M, Zhang Z, He L, Li Y (2021). Sarcopenia prevalence and associated factors among older Chinese population: findings from the China health and retirement longitudinal study. PLoS One.

[10] Alferink LJ, Trajanoska K, Erler NS (2019). Nonalcoholic fatty liver disease in the rotterdam study: about muscle mass, sarcopenia, fat mass, and fat distribution. J Bone Miner Res.

[11] Zhang X, He Z, Si Q (2022). The association of sarcopenia and visceral obesity with lean nonalcoholic fatty liver disease in Chinese patients with type 2 diabetes mellitus. J Diabetes Res.

[12] Lien YH (2017). Looking for sarcopenia biomarkers. Am J Med.

[13] Schutte JE, Longhurst JC, Gaffney FA, Bastian BC, Blomqvist CG (1981). Total plasma creatinine: an accurate measure of total striated muscle mass. J Appl Physiol Respir Environ Exerc Physiol.

[14] Huh JH, Choi SI, Lim JS, Chung CH, Shin JY, Lee MY (2015). Lower serum creatinine is associated with low bone mineral density in subjects without overt nephropathy. PLoS One.

[15] Weinberg EM, Trinh HN, Firpi RJ (2021). Lean Americans with nonalcoholic fatty liver disease have lower rates of cirrhosis and comorbid diseases. Clin Gastroenterol Hepatol.

[16] Hannemann A, Friedrich N, Dittmann K (2011). Age- and sex-specific reference limits for creatinine, cystatin C and the estimated glomerular filtration rate. Clin Chem Lab Med.

[17] Chalasani N, Younossi Z, Lavine JE (2018). The diagnosis and management of nonalcoholic fatty liver disease: practice guidance from the American Association for the Study of Liver Diseases. Hepatology.

[18] Pang JX, Pradhan F, Zimmer S (2014). The feasibility and reliability of transient elastography using Fibroscan®: a practice audit of 2335 examinations. Can J Gastroenterol Hepatol.

[19] Pavlov CS, Casazza G, Nikolova D, Tsochatzis E, Burroughs AK, Ivashkin VT, Gluud C (2015). Transient elastography for diagnosis of stages of hepatic fibrosis and cirrhosis in people with alcoholic liver disease. Cochrane Database Syst Rev.

[20] Abcar AC, Chan L, Yeoh H (2004). What to do for the patient with minimally elevated creatinine level?. Perm J.

[21] Kam LY, Huang DQ, Teng ML (2022). Clinical profiles of Asians with NAFLD: a systematic review and meta-analysis. Dig Dis.

[22] Costanzo M, Caterino M, Sotgiu G, Ruoppolo M, Franconi F, Campesi I (2022). Sex differences in the human metabolome. Biol Sex Differ.

[23] Wang YM, Zhu KF, Zhou WJ (2021). Sarcopenia is associated with the presence of nonalcoholic fatty liver disease in Zhejiang Province, China: a cross-sectional observational study. BMC Geriatr.

[24] Koo BK, Kim D, Joo SK (2017). Sarcopenia is an independent risk factor for non-alcoholic steatohepatitis and significant fibrosis. J Hepatol.

[25] Chun HS, Kim MN, Lee JS (2021). Risk stratification using sarcopenia status among subjects with metabolic dysfunction-associated fatty liver disease. J Cachexia Sarcopenia Muscle.

[26] Jocken JW, Goossens GH, Boon H (2013). Insulin-mediated suppression of lipolysis in adipose tissue and skeletal muscle of obese type 2 diabetic men and men with normal glucose tolerance. Diabetologia.

[27] Jocken JW, Langin D, Smit E (2007). Adipose triglyceride lipase and hormone-sensitive lipase protein expression is decreased in the obese insulin-resistant state. J Clin Endocrinol Metab.

[28] Mayerson AB, Hundal RS, Dufour S (2002). The effects of rosiglitazone on insulin sensitivity, lipolysis, and hepatic and skeletal muscle triglyceride content in patients with type 2 diabetes. Diabetes.

[29] Yao VJ, Sun M, Rahman AA, Samuel Z, Chan J, Zheng E, Yao AC (2021). Comparative analysis of metabolic risk factors for progression of non-alcoholic fatty liver disease. Clin Exp Hepatol.

